# Germination Shifts of C_3_ and C_4_ Species under Simulated Global Warming Scenario

**DOI:** 10.1371/journal.pone.0105139

**Published:** 2014-08-19

**Authors:** Hongxiang Zhang, Qiang Yu, Yingxin Huang, Wei Zheng, Yu Tian, Yantao Song, Guangdi Li, Daowei Zhou

**Affiliations:** 1 Northeast Institute of Geography and Agroecology, Chinese Academy of Sciences, Changchun, China; 2 State Key Laboratory of Forest and Soil Ecology, Institute of Applied Ecology, Chinese Academy of Sciences, Shenyang, China; 3 Graham Centre for Agricultural Innovation, Wagga Wagga, New South Wales, Australia; 4 Animal Science and Technology College, Jilin Agricultural University, Changchun, China; 5 College of Environment and Resources, Dalian Nationalities University, Dalian, China; Universidade Federal de Vicosa, Brazil

## Abstract

Research efforts around the world have been increasingly devoted to investigating changes in C_3_ and C_4_ species' abundance or distribution with global warming, as they provide important insight into carbon fluxes and linked biogeochemical cycles. However, changes in the early life stage (e.g. germination) of C_3_ and C_4_ species in response to global warming, particularly with respect to asymmetric warming, have received less attention. We investigated germination percentage and rate of C_3_ and C_4_ species under asymmetric (+3/+6°C at day/night) and symmetric warming (+5/+5°C at day/night), simulated by alternating temperatures. A thermal time model was used to calculate germination base temperature and thermal time constant. Two additional alternating temperature regimes were used to test temperature metrics effect. The germination percentage and rate increased continuously for C_4_ species, but increased and then decreased with temperature for C_3_ species under both symmetric and asymmetric warming. Compared to asymmetric warming, symmetric warming significantly overestimated the speed of germination percentage change with temperature for C_4_ species. Among the temperature metrics (minimum, maximum, diurnal temperature range and average temperature), maximum temperature was most correlated with germination of C_4_ species. Our results indicate that global warming may favour germination of C_4_ species, at least for the C_4_ species studied in this work. The divergent effects of asymmetric and symmetric warming on plant germination also deserve more attention in future studies.

## Introduction

Climate change may strongly shift the distribution of C_3_ and C_4_ vegetation. C_3_ and C_4_ distribution helps to estimate global vegetation primary production and carbon uptake by the terrestrial biosphere [1]. Many previous examinations of the distribution of C_3_ and C_4_ grasses have attempted to predict changes due to the effect of rising atmospheric CO_2_ concentration, and have suggested that the direct and indirect effects are likely to push C_3_/C_4_ relative abundances in opposite directions [2], [3]. A recent study indicates that C_4_ species tend to spread toward more northern latitudes and higher altitudes in the Inner Mongolia grassland. This was mainly triggered by increasing temperature, which overwhelmed the positive effect of rising CO_2_ concentrations on C_3_ species [4]. Others have found that temperature is the primary driver of C_4_ grass species distribution [5] and the aboveground productivity ratios of C_3_ and C_4_ plants [6], [7]. Long-term data sets from the shortgrass steppe have indicated that increased spring minimum temperature was correlated with decreased net primary production by the dominant C_4_ grass (*Bouteloua gracilis*) and increased abundance and production by exotic and native C_3_ forbs [8].

Global warming has advanced the yearly first-flowering times of plants [9]. This may change reproductive output and seed maturation time, which affects seed germination [10]. Germination percentage and rate of C_4_ species increased with temperature until 35°C to 40°C, while germination of C_3_ species was favored by cooler conditions [11]. Different germination responses of C_3_ and C_4_ species may affect ecosystem structure and functioning via species' altered relative competitive ability and/or net primary productivity. However, there has been little research on the consequences of global warming for plant seed germination [12], let alone for the effects on germination of C_3_ and C_4_ species.

Global warming has been found to be asymmetric [13], i.e. there are greater increases in daily minimum than maximum temperatures, resulting in declining diurnal temperature ranges [14], [15]. This pattern has been empirically demonstrated in several regions [16], [17]. To date, however, most modelling efforts and experimental manipulations investigating plant or ecosystem responses to climate change have assumed that future warming will occur primarily during the day or uniformly over the diurnal cycle [18], [19]. Only a few researchers have studied the effect of nocturnal temperature elevation on ecosystem functions [20]–[22]. To our knowledge, there is no study that has investigated the effect of asymmetric warming on plant regeneration from seed.

Predicting germination timing changes of C_3_ and C_4_ species under long term asymmetric warming compared with symmetric warming helps to elucidate C_3_ and C_4_ vegetation distribution changes. A thermal time approach can be used to model these aspects, though no one has attempted this before, except in modelling dormancy change [23] and seed germination [24], as well as seedling emergence [25] in current climate.

Previous research on germination response to alternating temperature has mainly focused on how seasonal changes in temperature regulate seed dormancy and germination [26]–[28] or on the effect of temperature fluctuations on germination [29]–[31]. In the current study, we alternated temperature regimes to simulate asymmetric and symmetric global warming and analyzed changes in germination patterns of C_4_ and C_3_ species using a thermal time model. The main objectives of this study were: (1) to determine the comparative effects of global warming on seed germination of C_3_ and C_4_ species; and (2) to compare the different effects on germination resulting from asymmetric versus symmetric warming.

## Materials and Methods

### Plant materials and seed collection

Six wild species were selected in this study, of which *Cynanchum chinense* R. Br., *Lappula myosotis* V. Worf., and *Saussurea amara* DC. were C_3_ species, and *Amaranthus retroflexus* L., *Portulaca oleracea* L., and *Echinochloa crusgalli* (L.) Beauv. were C_4_ species. These species are widely distributed in northern China. Mature seeds of the six target species were collected during autumn 2006 from wild populations in the Songnen grassland of China (sites near 44^o^40' N, 123^o^44' E), and stored in cloth bags at 4°C. The seeds were permitted to be collected around the land of the Grassland Ecosystem Experimental Station of Northeast Normal University. No protected species were sampled.

### Temperature regimes

To simulate global asymmetric and symmetric warming, two alternating temperature regimes were established (Table 1): a) The differential rate of warming between maximum and minimum temperatures (asymmetric warming, AW). Under this scenario, minimum/maximum temperatures were incrementally increased by +6°C/+3°C for each treatment from 5°C/20°C to 35°C/35°C. As a result, the mean temperature increased by 4.5°C and diurnal temperature range (DTR) decreased by 3°C for each set of treatments, with 6 treatments total in this regime; b) The same rate of warming between maximum and minimum temperatures (symmetric warming, SW). Under this scenario, both minimum and maximum temperatures increased by 5°C for each treatment from 5°C/15°C to 30°C/40°C. As a result, the mean temperatures incrementally increased by 5°C while the DTR remained constant at 10°C, with 6 treatments total in this regime. To test temperature metrics effects, two additional temperature regimes were established (Table 1): c) Minimum temperatures increased from 5°C to 35°C with 3°C increments, but the maximum temperature remained constant at 35°C across treatments (TmaxC). Effectively, the mean temperature increased by 1.5°C and the DTR decreased by 3°C across treatments, with 11 treatments total in this regime; d) minimum temperatures increased from 5°C to 20°C in 3°C increments, but maximum temperatures decreased from 35°C to 20°C in 3°C decrements (TaveC). Effectively, the mean temperature remained constant at 20°C, while the DTR decreased by 6°C across treatments, with 6 treatments total in this regime. To complete statistical analysis of thermal time model parameters, germination at 5–40°C constant temperatures with 5°C interval was also tested, with 8 treatments total in this regime. Together, 37 temperature regimes were established in this study. The DTR is defined as the difference between the diurnal maximum and minimum temperature. Alternating temperatures can be dissected into minimum temperature, maximum temperature and the calculated DTR and average temperature.

**Table 1:** The alternating temperature regimes used in the study.

Maximum temperature constant (TmaxC)	Average temperature constant (TaveC)	Asymmetric warming (AW)	Symmetric warming (SW)
TmaxC1: 5/35 (20, 30)*	TaveC1: 5/35 (20, 30)	AW1: 5/20 (12.5, 15)	SW1: 5/15 (10, 10)
TmaxC2: 8/35 (21.5, 27)	TaveC2: 8/32 (20, 24)	AW2: 11/23 (17, 12)	SW2: 10/20 (15, 10)
TmaxC3: 11/35 (23, 24)	TaveC3: 11/29 (20, 18)	AW3: 17/26 (21.5, 9)	SW3: 15/25 (20, 10)
TmaxC4: 14/35 (24.5, 21)	TaveC4: 14/26 (20, 12)	AW4: 23/29 (26, 6)	SW4: 20/30 (25, 10)
TmaxC5: 17/35 (26, 18)	TaveC5: 17/23 (20, 6)	AW5: 29/32 (30.5, 3)	SW5: 25/35 (30, 10)
TmaxC6: 20/35 (27.5, 15)	TaveC6: 20/20 (20, 0)	AW6: 35/35 (35, 0)	SW6: 30/40 (35, 10)
TmaxC7: 23/35 (29, 12)			
TmaxC8: 26/35 (30.5, 9)			
TmaxC9: 29/35 (32, 6)			
TmaxC10: 32/35 (33.5, 3)			
TmaxC11: 35/35 (35, 0)			

*The mean temperature and diurnal temperature range of each temperature regime were presented in parenthesis with comma between them.

### Germination test

Germination studies were conducted in growth chambers in August 2007. Each chamber corresponded to each temperature treatment. A 12-h photoperiod (Sylvania cool white fluorescent lamps, 25 µmol m^−2^ s^−1^, 400–700 nm) with 12-h dark period was maintained throughout the experiment. The temperatures cycled with half-hour linear transition periods between minimum and maximum temperatures (8:00–8:30 and 20:00–20:30, respectively).

Fifty seeds from each species were placed on two layers of filter paper in a grid (100 mm diameter) of plastic trays, and replicated 4 times within each temperature treatment. The filter paper was kept moistened with distilled water. Seeds were considered to have germinated when the radicle emerged. Germination status was recorded twice a day during the first 5 days, then once a day during the second 5 days. Germination was measured for 10 days because preliminary tests indicated that most of the germination events occurred within this period. The final germination percentage (GP) and germination rate (GR, the reciprocal of germination time) of each species were calculated with respect to the cumulative germination curves of each temperature treatment.

### Thermal time model theory

Garcia-Huidobro et al. [32] presented a model with two equations relating thermal time (degree days above a base temperature) to germination rate (the reciprocal of the time taken for a given fraction of seed to germinate) at a constant temperature. The two equations of the thermal time model (TT model) are:
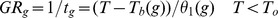


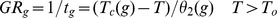



For any given subpopulation *g*, germination rate GR can be described by two straight lines. The slopes of the two lines are *θ*_1_(*g*) and *θ*_2_(*g*) (thermal time constant at suboptimal and supra-optimal temperatures) with the intersection of the two lines defined as *T*_o_ (optimum temperature, at which maximum germination rate occurs). The two points where germination percentages equal zero are defined as *T_b_*(*g*) and *T_c_*(*g*) (the minimum and maximum temperature, below or above which no germination occurs). The parameters are useful for field predictions and can be used to compare germination in different species, climates, and locations.

If alternating temperatures were all above or all below the optimum temperature, then the formulas were no different to those for constant temperature. However, if the temperature fluctuated from below the optimum to above, then the equation changed [33]. In such cases, the predicted germination rate for any subpopulation *g* is:
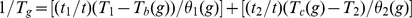
where *t*_1_ is the time below *T*_o_, with mean temperature of *T*_1_, and *t*_2_ is the time above *T*_o_, with mean temperature *T*_2_.

### Data analysis

Data were analysed using SPSS (version 13.0, SPSS Inc., Chicago, Illinois, USA). Germination percentage and germination rate of C_3_ or C_4_ species were the average of the three species with the same photosynthetic type. The effects of different alternating temperature treatments in each regime and the plant photosynthesis type (C_3_ and C_4_) on germination percentage and germination rate were examined using two-way ANOVA. Germination times for 1 percent seed (1% germination) were calculated from the cumulative germination curves in AW and SW temperature regimes for the six species and converted to rates in this study, which represents the initiative germination time of each batch of seeds. This standard (1% germination) was used instead of 50% germination because seed germination of certain species was less than 50% for some temperature treatments (see Figure S1). Thermal time model (TT model) parameters were analyzed using repeated probit regression as described previously [33]. The average daily temperatures were used for determining alternating temperatures. The model parameter (*T*_b_ and *θ*_1_) differences between C_3_ and C_4_ species, or between AW and SW temperature regimes within one photosynthetic type, were tested by Paired-Samples T-tests (2-tailed). The relationships between germination percentages/rates and the average temperatures in AW and SW regimes were fitted by either linear 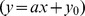 or quadratic regressions 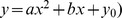 using SigmaPlot (version 10.0, Systat Software Inc., Richmond, California, USA), with 95% confidence of the fitted lines given. The differences in the slope (*a* in the equation) of the regression between AW and SW regimes for germination of C_4_ species were tested using the SMATR package in R software [34]. Pearson correlation analysis was carried out to test the correlation between GP/GR and temperature metrics (minimum, maximum, DTR, average) from the four alternating temperature regimes, totally 29 treatments.

## Results

### Germination responses to different temperature regimes

The interaction of plant photosynthetic type and temperature treatment had significant effects on germination percentage (GP) and germination rate (GR), except in the case for GR under TmaxC and TaveC temperature regimes (Table 2).

**Table 2:** The two-way ANOVA analysis of the effects of plant photosynthetic type (PPT) and temperature treatments (T) in four temperature regimes on germination percentage and germination rate.

	PPT (df = 1)	T (df = 10† or 5)	PPT * T (df = 10† or 5)
GP			
TmaxC	32.24***	1.24ns	2.38*
TaveC	0.13ns	0.30ns	3.23*
SW	0.43ns	0.88ns	3.18*
AW	0.05ns	0.19ns	3.40*
GR			
TmaxC	557.85***	5.37**	1.02ns
TaveC	<0.01ns	0.07ns	1.56ns
SW	1.05ns	1.11ns	14.16***
AW	0.84ns	1.18ns	4.97**

ns, *P*>0.05;

*, *P*<0.05;

**, *P*<0.01;

***, *P*<0.001

† The degree of freedom for TmaxC temperature regime is 10 and df of other temperature regimes is 5.

**Figure 1:**
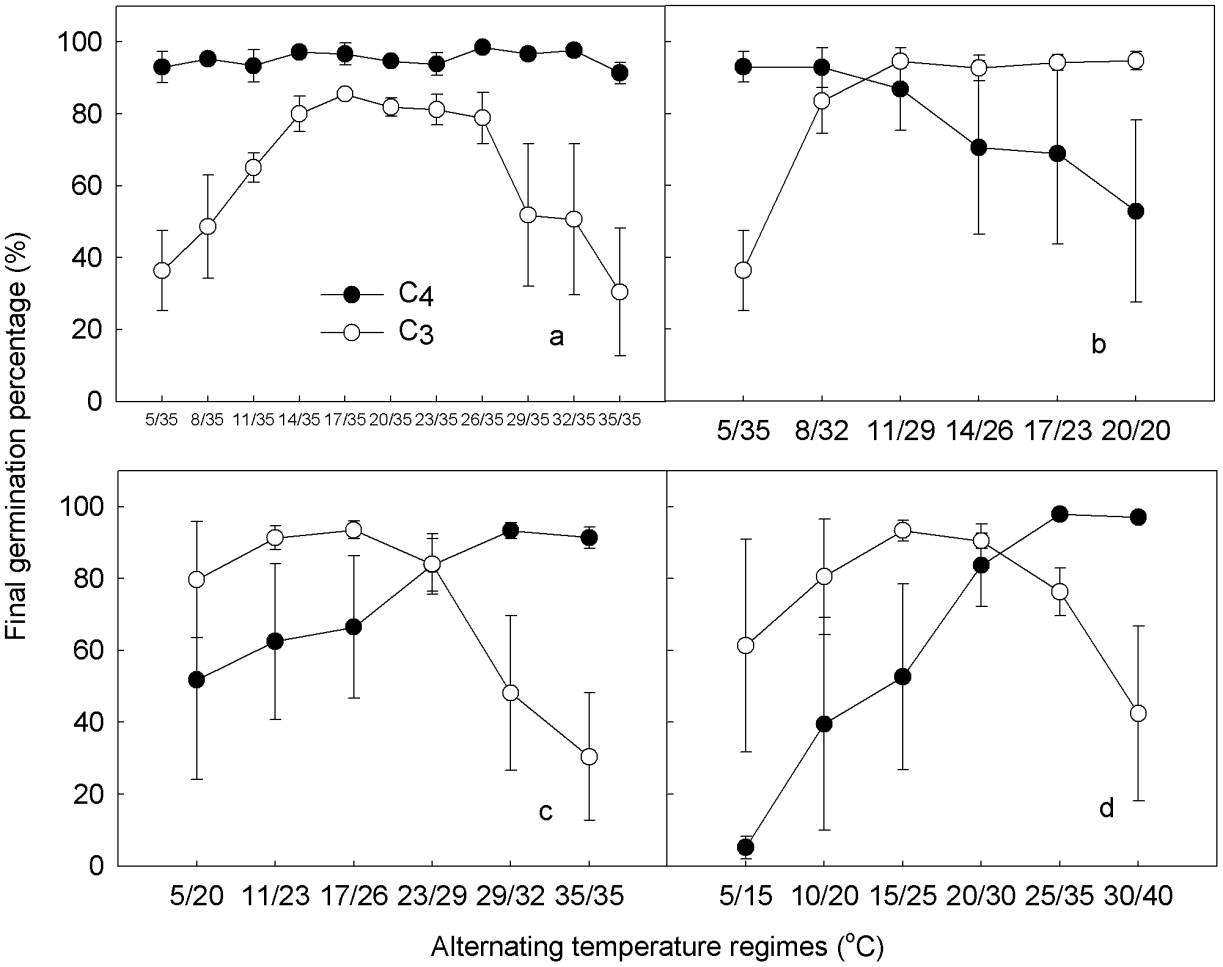
Germination percentages of C_4_ (closed circles, average of *E. crusgalli*, *P. oleracea* and *A. retroflexus*) and C_3_ species (open circles, average of *L. myosotis*, *S*. *amara* and *C*. *chinense*) under different alternating temperature regimes (a, TmaxC; b, TaveC; c, AW; d, SW). Mean ± S.E.

GP of C_4_ species were all above 90% in the TmaxC temperature regime, while GP of C_3_ species increased linearly from 5/35°C to 17/35°C and remained around 80%–85% from 17/35°C to 26/35°C, then decreased sharply as the minimum temperature increased thereafter (Figure 1a). In the TaveC temperature regime, GP of C_4_ species decreased as the minimum temperature increased, while GP of C_3_ species increased, reached its highest value at 11/29°C, and remained subsequently constant (Figure 1b). In the AW and SW temperature regimes, GP of C_4_ species increased, whereas GP of C_3_ species increased and then decreased with the increasing temperatures (Figure 1c, d).

**Figure 2:**
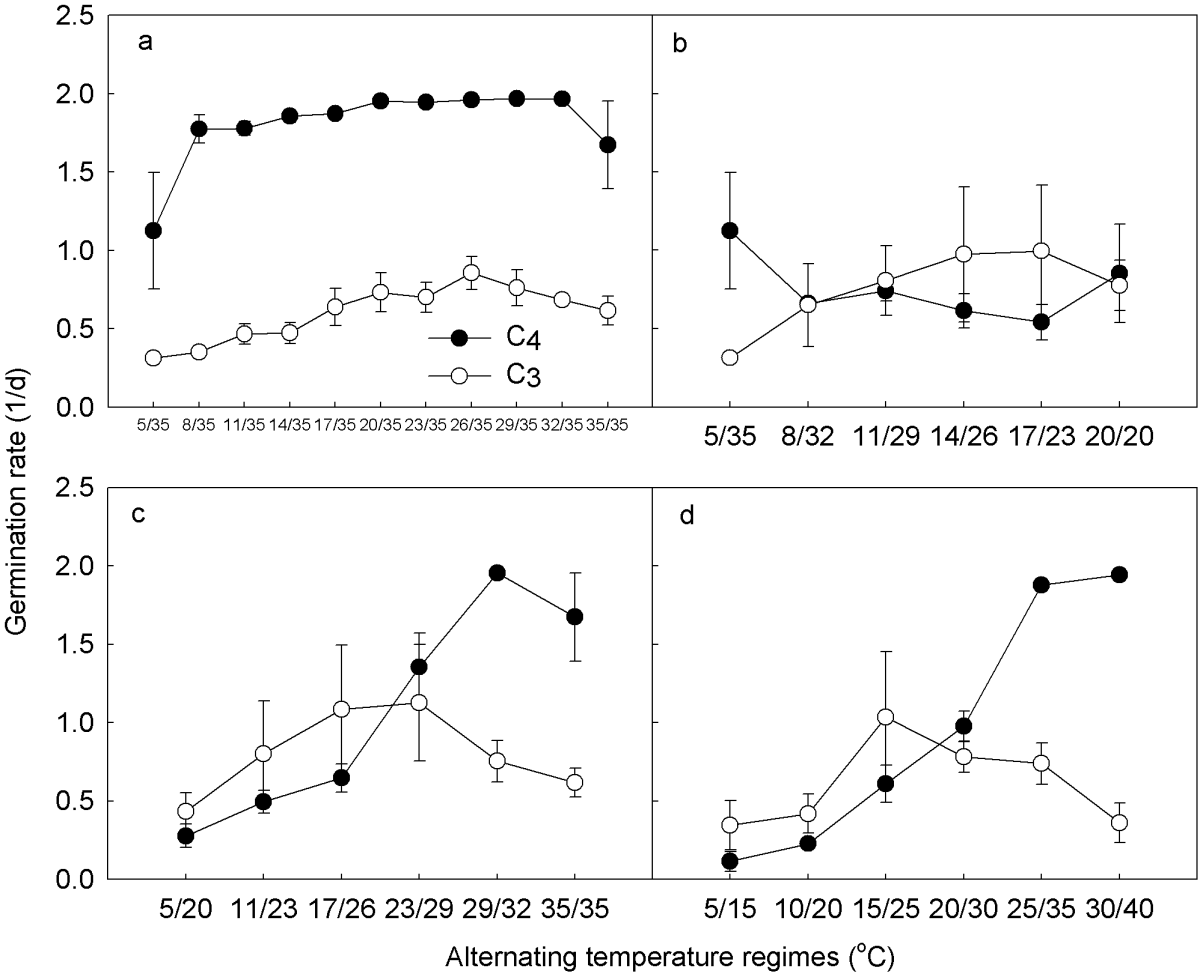
Germination rates of C_4_ and C_3_ species under different alternating temperature regimes. See Fig. 1 for symbols.

In the TmaxC temperature regime, GR of C4 species at 5/35°C was significantly lower than those at the other altered temperatures (*P*<0.05, Figure 2a). As the minimum temperature increased, GR of C_3_ species increased gradually and reached peak at 26/35°C, and then decreased. In the TaveC temperature regime, GR of C_4_ species tended to decrease with the increasing minimum temperature, while GR of C_3_ species tended to increase until 17/23°C (Figure 2b). However, there were no significant differences in all values except GR at 5/35°C between C_4_ and C_3_ species (*P*<0.05). In the AW and SW temperature regimes, GR of C_4_ species increased until 30°C of average temperature (29/32°C for AW or 25/35°C for SW), while GR of C_3_ species increased and then decreased with the increasing temperatures (Figure 2c, d). GR of the three C_3_ and C_4_ species responded consistently to the temperature regimes, with only magnitude difference (Figure S2).

### Comparison of TT model parameters and germination under asymmetric and symmetric warming

**Figure 3:**
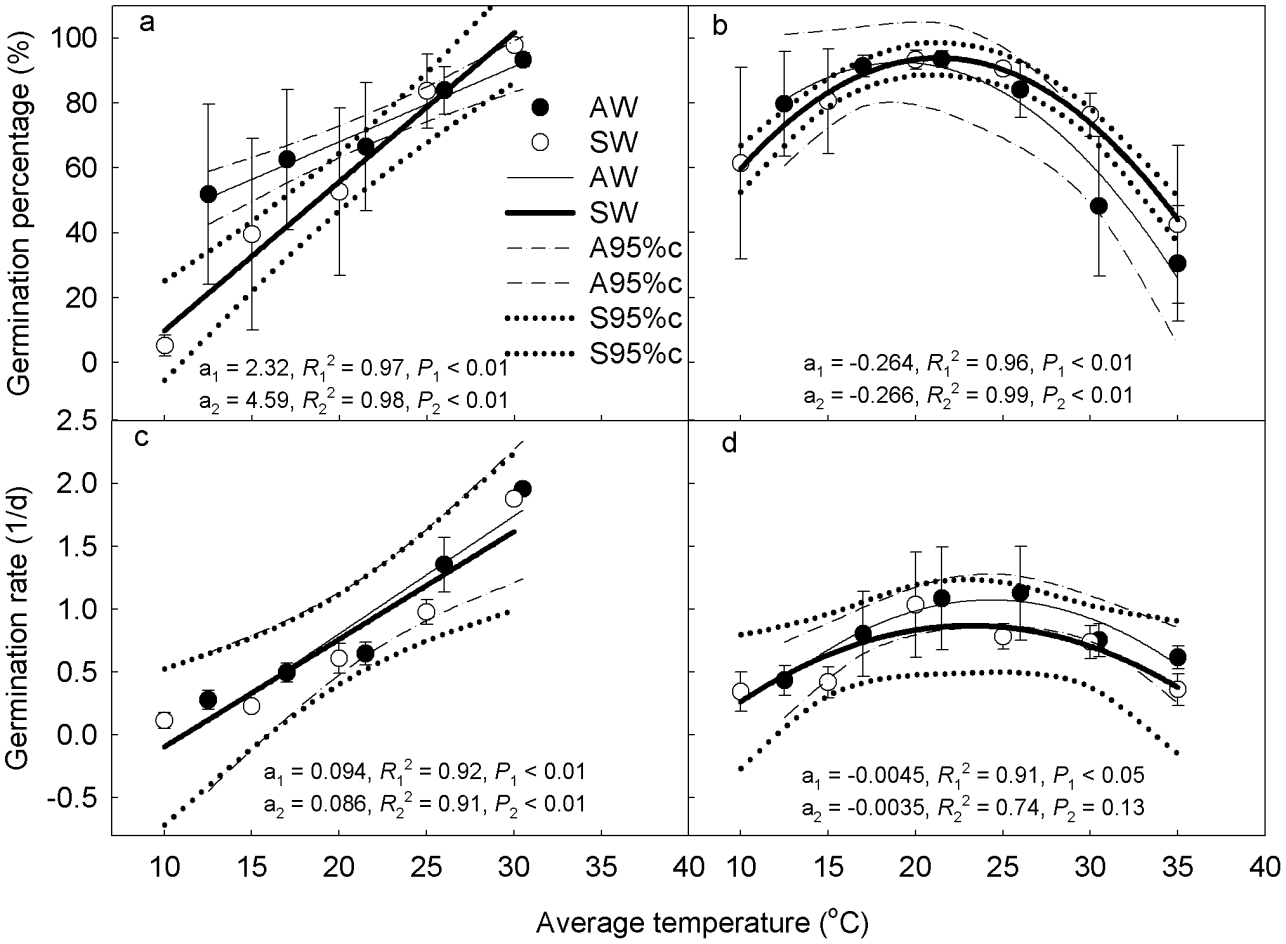
Germination percentage (a, b) and germination rate (c, d) changes of C_4_ (a, c) and C_3_ (b, d) species with increasing average temperature in the AW and SW temperature regimes. The relationship between C_4_ species and the average temperature is fitted by linear equation; the relationship between C_3_ species and the average temperature is fitted by polynomial equation. Dash-dot lines represent 95% confidence of AW temperature regime and dotted lines represent 95% confidence of SW temperature regime. a_1_, *R*_1_^2^, *P*_1_ represent the parameter, coefficient of determination and probability for the fitting in the AW regime and a_2_, *R*_2_^2^, *P*_2_ represent the parameter, coefficient of determination and probability for the fitting in the SW regime.

For all species in the AW and SW alternating temperature regimes (except C_3_ species *L. myosotis* in the SW temperature regime), the linear regressions of the TT model explained more than 87% variation (Table 3). For the three C_4_ species and the average, the estimated *T_b_* of 1% germination in the AW temperature regime was higher than those in the SW temperature regime (*P* = 0.059). However, the estimated *θ*_1_ in the AW regime were lower than those in the SW regime (*P* = 0.057). Therefore, the germination rates were difficult to compare between the two temperature regimes according to the TT model equation (Figure 3c). For *C. chinense* and the average of three C_3_ species, *T_b_* and *θ*_1_ of 1% germination in the AW temperature regime were both lower than that in the SW temperature regime (*P* = 0.891 for *T_b_*, *P* = 0.081 for *θ*_1_), such that germination rates of the C_3_ species in the AW temperature regime were higher than values in the SW temperature regime (Figure 3d). Compared with C_3_ species, *T_b_* of C_4_ species were significantly higher in both the AW and SW temperature regimes (*P*<0.05). If C_3_ species *L. myosotis* is excluded, *θ*_1_ of C_4_ species was also significantly lower than C_3_ species in both temperature regimes (*P*<0.05).

**Table 3:** Thermal time model parameter estimates (*T_b_*, minimum temperature; *θ*_1_, thermal time constant) for C_4_ and C_3_ species under the symmetric warming (SW) and asymmetric warming (AW) alternating temperature regimes.

		*T_b_* (°C)	*θ*_1_ (°C•d)	*R^2^*	*P*
C_4_ species					
*P. oleracea*	SW	9.6	12.4	0.94	0.0013
	AW	10.4	11.3	0.94	0.0015
*E. crusgalli*	SW	10.4	12.3	0.95	0.0011
	AW	10.7	10.0	0.93	0.0079
*A. retroflexus*	SW	12.6	11.7	0.92	0.0028
	AW	13.3	10.5	0.87	0.0206
C_4_ species average	SW	10.9	12.1		
	AW	11.5	10.6		
C_3_ species					
*L. myosotis*	SW	6.3	8.2	0.76	0.3275
	AW	7.2	7.3	0.97	0.1123
*S. amara*	SW	1.7	34.6	0.93	0.0349
	AW	1.8	31.8	0.95	0.1372
*C. chinense*	SW	5.6	28.6	0.90	0.0133
	AW	4.3	25.5	0.94	0.0057
C_3_ species average	SW	4.5	23.8		
	AW	4.4	21.5		

For relationship between germination percentage and the average temperature, the parameter *a* in the AW regime was smaller than that in the SW regime, regardless of photosynthetic type (Figure 3a, b) and the difference was significant for C_4_ species (*P*<0.01). This suggests that changes were more intense under symmetric warming (SW) than asymmetric warming (AW), especially for C_4_ species. For relationship between germination rate and the average temperature, the parameter *a* in the AW regime was larger than that in SW regime for both C_4_ and C_3_ species. However, the parameter *a* between the two temperature regimes was not significantly different for C_4_ species (*P* = 0.74, Figure 3c) and the linear regression between germination rate and average temperature for C_3_ species in the SW regime was not significant (*P* = 0.13, Figure 3d).

### Correlation between GP/GR and temperature metrics

For C_4_ species average, GP/GR were positively correlated with minimum, maximum and average temperature (*P*<0.05, Table 4), with the highest correlation coefficients between GP/GR and maximum temperature (*r* = 0.939 or *r* = 0.876). GRs of C_4_ species were negatively correlated with DTR, though it was not significant (*P* = 0.798). All of the three C_4_ species responded similarly. For C_3_ species however, GPs were negatively correlated with maximum and average temperatures (*P*<0.05), and GRs were negatively correlated with DTR (*P*<0.01). Furthermore, the results of the three C_3_ species were inconsistent.

**Table 4:** Pearson correlation analysis of germination percentage (GP) and germination rate (GR) with temperature metrics (TM, minimum, maximum, average, diurnal temperature range (DTR)) from the four alternating temperature regimes for C_4_ and C_3_ species (*r*, correlation coefficient; *P*, probability for the correlation).

Species	TM	GP		GR	
		*r*	*P*	*r*	*P*
C_4_ species					
*P. oleracea*	Minimum	0.388	0.037	0.590	0.001
	Maximum	0.870	<0.001	0.863	<0.001
	DTR	0.221	0.250	−0.005	0.981
	Average	0.678	<0.001	0.815	<0.001
*E. crusgalli*	Minimum	0.200	0.298	0.624	<0.001
	Maximum	0.434	0.019	0.822	<0.001
	DTR	0.103	0.594	−0.072	0.712
	Average	0.343	0.069	0.819	<0.001
*A. retroflexus*	Minimum	0.462	0.012	0.636	<0.001
	Maximum	0.958	<0.001	0.846	<0.001
	DTR	0.206	0.285	−0.067	0.728
	Average	0.771	<0.001	0.839	<0.001
C_4_ species average	Minimum	0.439	0.017	0.640	<0.001
	Maximum	0.939	<0.001	0.876	<0.001
	DTR	0.216	0.260	−0.050	0.798
	Average	0.746	<0.001	0.855	<0.001
C_3_ species					
*L. myosotis*	Minimum	−0.416	0.025	−0.002	0.992
	Maximum	−0.679	<0.001	−0.440	0.017
	DTR	−0.049	0.801	−0.324	0.087
	Average	−0.608	<0.001	−0.208	0.278
*S. amara*	Minimum	−0.576	0.001	0.438	0.017
	Maximum	−0.594	0.001	−0.071	0.713
	DTR	0.189	0.326	−0.531	0.003
	Average	−0.679	<0.001	0.270	0.157
*C. chinense*	Minimum	0.539	0.003	0.802	<0.001
	Maximum	0.312	0.100	0.385	0.039
	DTR	−0.358	0.057	−0.590	0.001
	Average	0.521	0.004	0.737	<0.001
C_3_ species average	Minimum	−0.299	0.115	0.333	0.077
	Maximum	−0.514	0.004	−0.197	0.307
	DTR	−0.055	0.777	−0.509	0.005
	Average	−0.449	0.015	0.138	0.474

## Discussion

Germination percentage and germination rate of C_4_ species increased linearly under the AW regime (Figure 1c and 2c), while those of C_3_ species increased and then decreased with the increasing temperature. This suggests that long term global asymmetric warming may favor seed germination of C_4_ species, at least for the tested species, which is consistent with the model prediction by Thorpe et al. [35] and inference from Sage and Kubien [36]. Spring events are changing more than autumn events as they are more sensitive to climate and are also undergoing the greatest alterations of climate relative to other seasons [37]. Germination and emergence are mainly spring events in most temperate regions [38], which are important research topics related to global warming. Though we just studied three C_3_ and three C_4_ species and more research is needed on this topic, our results somehow reflected the trends of C_3_ and C_4_ species germination and emergence shift under global warming.

In our study, symmetric and asymmetric warming had different impacts on germination of C_4_ species. The symmetric warming treatments significantly overestimated the speed of germination percentage change with temperature (*P*<0.05), compared to the asymmetric warming (Figure 3a). Another evidence is that the average base temperature is higher and the average thermal time constant is lower in the AW alternating temperature regime compared to the SW regime (Table 3), which is near significant (*P* = 0.059 for base temperature, *P* = 0.057 for thermal time constant).

Ecosystem warming studies have been performed for more than 20 years using a variety of methods including heat-resistance cables, infrared (IR) lamps, field chambers (e.g. OTC), and night-time warming. Historic air temperature data and most models suggest that much of the global warming increase will occur during the night-time hours. Therefore, artificial night-time warming is ideally suited for replicating a potentially relevant form of climate change [39]. Field night warming has been achieved by heating with IR at night-time [21], by reflective curtains covering the vegetation at night [40], or by light-weight aluminum fabric shelters (mounted on rollers similar to a window shade) that are drawn across the warming plots at night [41]. In this study, we first used alternating temperature regimes controlled by different growth chambers to simulate consecutive global asymmetric (+3/+6°C at day/night continuously) and symmetric (+5/+5°C at day/night continuously) warming (Table 2). This method can be used to predict the trend of shifts in seed germination and seedling growth under global warming.

Phillips et al. [42] suggest that it is significant to understand the influence of both day and night-time warming on the carbon balance of plants and concluded that changes in daily mean temperatures, rather than changes in minimum or maximum temperature, are sufficient for predicting ecosystem carbon fluxes in a Mediterranean grassland system. Another report indicates that a changing daily temperature regime may be important in determining plant responses to warming temperatures and should be considered in predictions of plant and ecosystem responses to future climate change [43]. The correlation between germination and the temperature metrics (minimum, maximum, diurnal temperature range (DTR) and average temperature) from the four temperature regimes for C_4_ and C_3_ species were analyzed in this study (Table 4). For C_4_ species, GP/GR was most correlated with the maximum temperature, while the results of the three C_3_ species were inconsistent. Von Fischer et al. [7] suggest that daily maximum temperature better predicts percent of C_4_ species than daily average or minimum temperature. Hattersley [44] also found summer (January) temperature in Australia had highest correlation with percent of C_4_ species. DTR is an important index of climate change [45]. In our study, average and minimum temperature was significantly correlated with germination of C_4_ species, but not C_3_ species. However, DTR was not correlated with germination, except with the germination rates of the C_3_ species *S. amara* and *C. chinense*.

## Conclusions

This work indicates that global symmetric and asymmetric warming favors seed germination of C_4_ species, rather than C_3_ species, according to the results of the tested species in this study. Compared to asymmetric warming, symmetric warming overestimated the speed of germination percentage change with temperature for C_4_ species. Asymmetric and symmetric warming had no significant effects on germination of C_3_ species. Among the temperature metrics (minimum, maximum, diurnal temperature range and average of the alternating temperature), maximum temperature (day-time temperature) was found to be most correlated with germination for C_4_ species. Germination of C_3_ species responded inconsistently to the temperature metrics. Using alternating temperature regimes controlled by different growth chambers to simulate consecutive global asymmetric and symmetric warming is a good method, which can be used to predict the trend of shifts in plant early growth under global warming.

## Supporting Information

Figure S1 Final germination percentages of six species under different alternating temperature regimes (TmaxC, a, b; TaveC, c, d; AW, e, f; SW, g, h). C_4_ species: a, c, e, g; C_3_ species: b, d, f, h.
(TIF)

Figure S2 Germination rates of six species under different alternating temperature regimes. See S1 for symbols.
(TIF)
